# Water quality index calculated from biological, physical and chemical attributes

**DOI:** 10.1007/s10661-014-4163-1

**Published:** 2014-12-10

**Authors:** Francisco Cleiton Rocha, Eunice Maia Andrade, Fernando Bezerra Lopes

**Affiliations:** The Engenharia Agrícola Department, Federal University of Ceará, 12.168, Centro de Ciências Agrárias. Endereço: Av. Mister Hull, 2977, Bloco 804, - Campus do Pici, Fortaleza, 60450-760 Ceará Brazil

**Keywords:** Macroinvertebrates, Biological Monitoring Working Party (BMWP), Biological metrics, Surface water, Semi-arid, Aquatic macrophytes

## Abstract

To ensure a safe drinking water supply, it is necessary to protect water quality. To classify the suitability of the Orós Reservoir (Northeast of Brazil) water for human consumption, a Water Quality Index (WQI) was enhanced and refined through a Principal Component Analysis (PCA). Samples were collected bi-monthly at seven points (P1 – P7) from July 2009 to July 2011. Samples were analysed for 29 physico-chemical attributes and 4 macroinvertebrate metrics associated with the macrophytes *Pistia stratiotes* and *Eichhornia crassipes*. PCA allowed us to reduce the number of attributes from 33 to 12, and 85.32 % of the variance was explained in five dimensions (C1 – C5). Components C1 and C3 were related to water-soluble salts and reflect the weathering process, while C2 was related to surface runoff. C4 was associated with macroinvertebrate diversity, represented by ten pollution-resistant families. C5 was related to the nutrient phosphorus, an indicator of the degree of eutrophication. The mean values for the WQIs ranged from 49 to 65 (rated as fair), indicating that water can be used for human consumption after treatment. The lowest values for the WQI were recorded at the entry points to the reservoir (P3, P1, P5, and P4), while the best WQIs were recorded at the exit points (P6 and P7), highlighting the reservoir’s purification ability. The proposed WQI adequately expressed water quality, and can be used for monitoring surface water quality.

## Introduction

Water is essential for sustaining life on Earth (Giriyappanavar and Patil 2013). However, it has been altered significantly from its natural state, and human activities can affect its availability for various uses, both in quantity and in quality (Magesh and Chandrasekar 2013). Faced with this reality, and coupled with the limited availability of freshwater for human consumption on our planet (Pal et al. 2013), the quality of the available water must be monitored. This is particularly important in arid and semi-arid regions (Aenab et al. 2012), which are characterised by irregular rainfall, both spatially and temporally, and with high rates of evapotranspiration (Andrade et al. 2010).

In tropical semi-arid regions, such as the northeastern part of Brazil where rivers are ephemeral or intermittent, reservoirs are the main source of water; monitoring their water quality is therefore essential. Water quality indices developed for temperate regions, based on physical and chemical attributes, have been used to assess water quality in this area (Santos et al. 2014; Batista et al. 2014). We believe, however, that further investigation is needed to obtain an improved understanding of the quality of the waters of the Orós reservoir.

Water quality monitoring is traditionally carried out by environmental agencies who analyse physical (temperature, pH and transparency), chemical (BOD, DO, total phosphorus, total ammonia, nitrate, calcium, magnesium, sodium, potassium, bicarbonate and phosphate) and microbiological attributes (total and faecal coliforms) (Vasanthavigar et al. 2010) of water. Water quality indices (WQIs) are tools that use an integrative methodology to convert a large set of data into a single number to express the water quality (Lumb et al. 2011); they can be calculated using physical, chemical and microbiological data collected by environmental agencies (Hurley et al. 2012).

WQIs are easier and quicker for the general public to understand than a large amount of complicated environmental data presented in reports. They can therefore be very useful in water resource and watershed management (Yisa and Jimoh 2010); they can also reduce the cost of analyses by highlighting attributes that are less important for water quality, thereby allowing us to omit them. Although WQIs have been used for many decades in other parts of the world, Brazilian researchers have only started to develop and apply them in the past decade (Almeida and Schwarzbold 2003; Andrade et al. 2005).

Generally, WQIs are prepared using physical and chemical attributes. However, Yan et al. (2014) have suggested that biological attributes obtained from studies of the structure of communities of organisms that act as bioindicators of water quality, such as macroinvertebrates, fish, macrophytes, phytoplankton and zooplankton, should be included. According to Baptista (2008), these organisms react to disturbances in the environments in which they live, whether of natural or anthropogenic origin. Additionally, Ferreira et al. (2011) observed that, when disruption is severe, the more resistant bioindicator organisms may become dominant, while the more sensitive become rare or absent.

In the last decade, bioindicators have been widely used in many countries, such as the UK, Spain, China, Australia, the USA and Canada (Morse et al. 2007). In the world, the USA is at a more advanced stage in the use of macroinvertebrates and other groups of organisms in water quality assessment (Hurley et al. 2012).

Brazilian legislation, by means of the National Policy for Water Resources (Law 9433/97) and Brasil (2001), provides for water quality to be assessed with biological indicators (Buss and Borges 2008). Current legislation, according to Oliveira et al. (2008), represents an important advance, but biomonitoring of waterbodies is still not mandatory. Brazilian environmental laws and regulatory processes only require that water quality assessments are based on physical, chemical and bacteriological parameters. As a result, macroinvertebrate metrics have only been used in the recent past as bioindicators of water quality in Brazil, especially in the Amazon region (Silveira et al. 2005; Uherek and Gouveia 2014).

Traditionally, physical, chemical and biological indicators have been treated separately by WQIs. Also, to date, few indices have been proposed for the reservoirs in this semi-arid region of Brazil. One noteworthy example is the index proposed by Andrade et al. (2005), in which only chemical attributes were considered. Using a cross-sectional view of the processes that determine water quality, the aim of this study therefore was to develop a WQI for the waters of artificial reservoirs, which would consider the physical and chemical attributes and the biological metrics of macroinvertebrates of a semi-arid tropical region. As a result of this study, we will have a new method for evaluating water quality, in which physical, chemical and biological attributes will be integrated in a single WQI.

## Materials and methods

### Description of the study area

This study was carried out in the Orós reservoir, which is in the watershed of the Upper Jaguaribe River, in the semi-arid region of the state of Ceará, Brazil (6° 8′ 3″ S–6° 20′ 26″ S and 38° 54′ 56″ W–39° 13′ 28″ W). The reservoir has a total water storage capacity of 1.94 billion m^3^, and a contributing area of 25,000 km^2^ (DNOCS 2014).

Using the Köppen classification, the climate in the region is BSw'h', otherwise known as semi-arid hot with summer/autumn rains and a monthly average temperature greater than 18 °C. The average rainfall is 750 mm year^−1^, with a potential evaporation of 1988 mm year^−1^ and insolation of 2945 h year^−1^. Rainfall in the region is characterised by a high spatial and temporal variability, with the main limitation being the irregularity of the regime rather than the actual amount of annual rainfall.

The geology of the area is dominated by crystalline basement rocks with a predominance of homogeneous and heterogeneous migmatites, gneiss and quartzite (Radambrasil 1981). According to Embrapa (2006), the soils of the watershed fall into seven classes, with neosols (31.9 %) and argisols (29.06 %) being the most prevalent.

The resident population in the Upper Jaguaribe watershed is approximately 600,000 inhabitants. On average, 85.95 % of households have a piped water supply; however, only 11.22 % are connected to a sewage system (IPECE 2012).

**Fig. 1:**
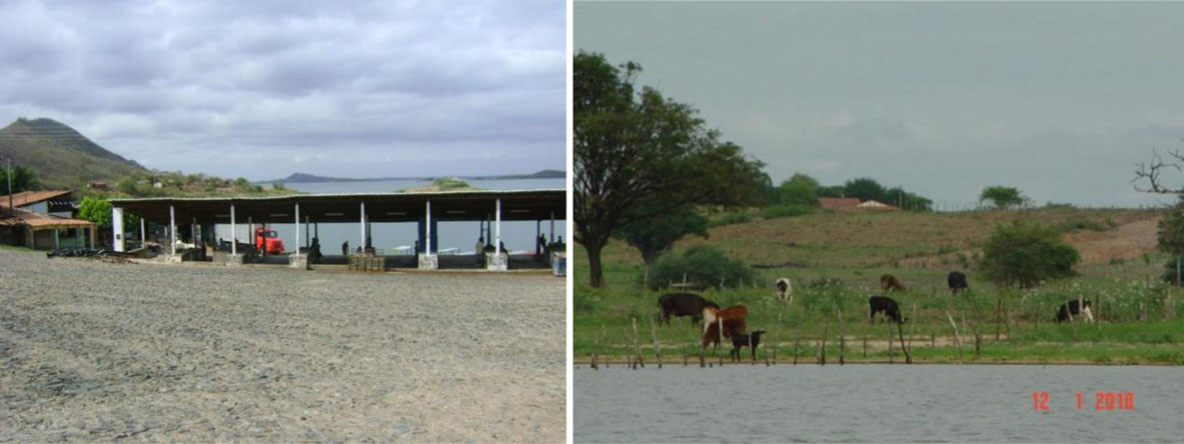
Fish farming and livestock around the Orós reservoir

Agriculture, livestock and manufacturing are the main sources of income (Lopes et al. 2014). Fish farming and subsistence farming have been developed by communities located around the Orós reservoir (Fig. 1). Batista et al. (2014) state that 42.38 % of the permanent farmland area is used for pasture, 24.05 % for poultry farming, 2.08 % for maize cultivation, 15.83 % for rice cultivation and 7.89 % for other crops and uses; only 7.77 % is covered by natural vegetation.

### Sampling and analysis

**Fig. 2:**
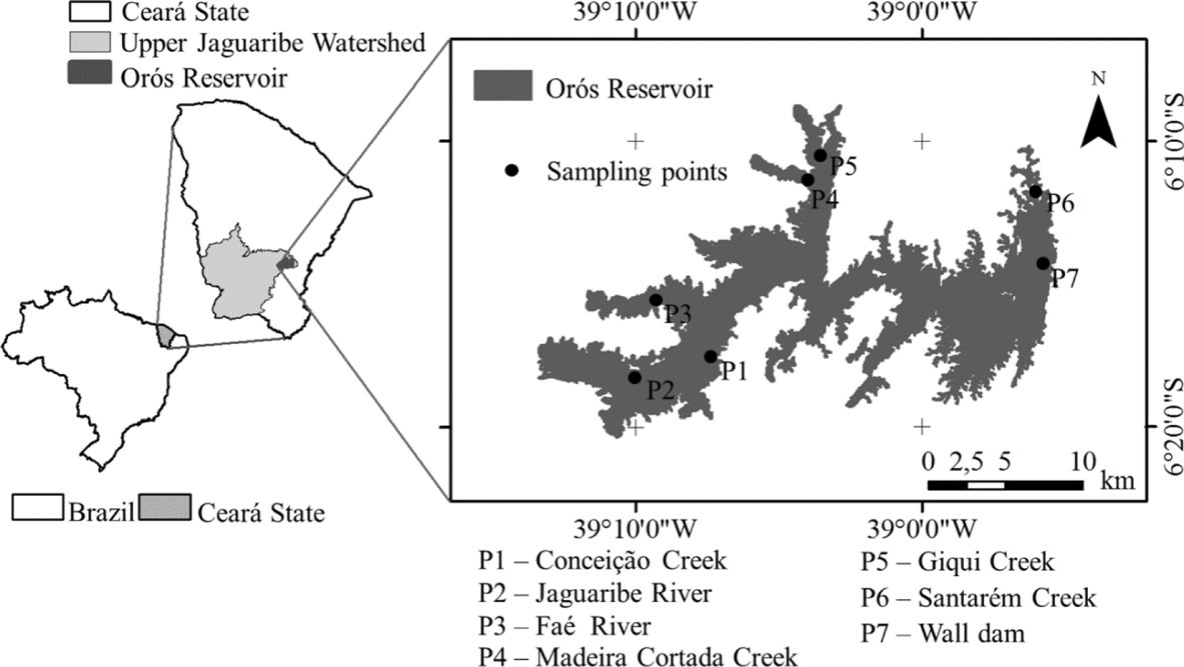
Geographical position of the reservoir and the locations of the sampling points

The data used to develop the proposed WQI were obtained from seven sampling sites (P1, Conceição; P2, River Jaguaribe; P3, River Faé; P4, Madeira Cortada; P5, Giqui; P6, Santarém; and P7, Upstream) in the Orós reservoir. Six of these sites correspond to the confluences of the major tributaries (P1 to P6), while the other site (P7) is located near the spillway of the reservoir, as shown in Fig. 2. All the sampling points were accurately georeferenced with a Garmin GPS. The sampling points were chosen so that the inputs from the six major tributaries to the reservoir were represented.

The water samples were taken at a depth of 30 cm from the surface using a range of specific containers: Samples to be analysed for phytoplankton were collected in 1-L flasks containing formalin and lugol; samples for dissolved oxygen determination were collected in separate flasks; and samples for the remaining attributes were collected in 1.5-L properly decontaminated bottles. The samples were placed into isothermal boxes and taken to the laboratory, where they were either processed immediately or properly stored. pH, temperature, Secchi transparency and electrical conductivity (EC) were measured in the field.

Sampling was carried out nine times from July 2009 to July 2011. Samples from each sampling point were tested for 33 attributes (physical, chemical, microbiological and biological) (Table 1), giving a total of 2772 tests (7 points × campaigns × 33 attributes).

**Table 1:** Attributes tested, methodologies and references

Attributes	Methodologies	References
Temperature (°C)	Mercury filament thermometer 0–60 °C	APHA (2005)
Turbidity (uT)	Turbidimetric	
Apparent colour (uH)	Colourimetric	
Electrical conductivity (dS m^−1^)	Conductivity meter	
Secchi transparency (m)	Secchi disk	
pH	pH meter	
Total solids, TS (mg L^−1^)	Drying at 103–105 °C	
Total volatile solids, TVS (mg L^−1^)	Ignition at 500–550 °C	
Total fixed solids, TFS (mg L^−1^)		
Total suspended solids, TSS (mg L^−1^)	Vacuum filtration with fibreglass membrane 0.45-μm porosity	
Total dissolved solids, TDS (mg L^−1^)	Drying at 103–105 °C	
Inorganic suspended solids, ISS (mg L^−1^)	Vacuum filtration with fibreglass membrane 0.45-μm porosity	
Volatile suspended solids, VSS (mg L^−1^)	Ignition at 500–550 °C	
Dissolved oxygen (mg L^−1^)	Winkler method (Azide modification)—iodometry	
DBO_5_ (mg L^−1^)	Standard flasks—iodometry	
Total phosphorus, TP (mg L^−1^)	Spectrophotometry—ascorbic acid	
Soluble orthophosphate, SOP (mg L^−1^)		
Sulphates (mg L^−1^)	Turbidimetric	
Chlorides (mg L^−1^)	Argentometric titration	
Potassium (mg L^−1^)	Photometry—flame emission	
Sodium (mg L^−1^)		
Calcium (mg L^−1^)	Titration	
Magnesium (mg L^−1^)		
*Escherichia coli* (NMP/100 mL)	Colilert	
Thermotolerant coliforms (NMP/100 mL)	Multiple tubes in an A1 medium	
Phytoplankton—qualitative and quantitative	Bright field microscopy of slides prepared from sediment obtained by centrifugation at 1500 rpm for 5–10 min to estimate the density of cyanobacteria and identification of phytoplankton using dichotomous identification keys	
Total ammonia nitrogen, TAN (mg L^−1^)	Spectrophotometric—macro-Kjeldahl distillation followed by direct Nesslerization	
Ammonia (mg L^−1^)		
Nitrate (mg L^−1^)	Spectrophotometric—sodium salicylate	Rodier (1975)
Chlorophyll *a* (μg L^−1^)	Spectrophotometric—hot extraction with methanol	Jones (1979)
ricF(ec)	Richness of macroinvertebrate families associated with the roots of the macrophyte *Eichhornia crassipes*	Brusca and Brusca (2007); Mugnai et al. (2010)
ricF(ps)	Richness of macroinvertebrate families associated with the roots of the macrophyte *Pistia stratiotes*	
Abundance (Abec)	Abundance of macroinvertebrates associated with the roots of the macrophyte *Eichhornia crassipes*	
Abundance (Abps)	Abundance of macroinvertebrates associated with the roots of the macrophyte *Pistia stratiotes*	

The testing frequency for water quality attributes is generally based on the population being supplied or on the volume of water distributed (CESTEB 2011). To ensure that the data were representative of the range of possible environmental conditions, the samples were collected in both the wet and dry seasons, following the standards of the National Water Quality Assessment Program (PNQA) in Brazil (ANA 2012).

In this study, the richness of the macroinvertebrate families associated with the roots of two aquatic macrophyte species, *Pistia stratiotes* and *Eichhornia crassipes*, was used to represent the biological attributes. At each of the seven sampling points, samples of *P. stratiotes* and *E. crassipes* were taken in triplicate. Samples were collected in a delta net with a mesh aperture of 500 μm and were stored in properly labelled plastic containers with hydrated ethyl alcohol (80 %).

In the laboratory, the macrophyte roots were washed to remove any macroinvertebrates. These were separated and fixed in hydrous ethyl alcohol (70 %) for later identification with the help of various identification keys (Brusca and Brusca 2007; Mugnai et al. 2010).

The richness of the invertebrate families was then calculated for each sampling point, and scores were allocated to the families based on the Biological Monitoring Working Party (BMWP) index proposed by Armitage et al. (1983) and later adapted by Mustow (2002), Wyżga et al. (2013) and others. This classification assigns scores to each family of macroinvertebrates that are present. The BMWP classifies organisms at the family taxonomic level and allocates values ranging from 1 to 10 (Alba-Tercedor et al. 2002). Families sensitive to high levels of organic pollutants receive higher values, while resistant families receive lower values (Rossaro et al. 2007).

### Selection of attributes

We used factor analysis/principal component analysis (FA/PCA) to identify the most important attributes in explaining the variability of water quality. There were four stages to this analysis:

(i) *Preparation of the correlation matrix*—used to eliminate the problem of using different scales and units to measure the attributes
(ii) *Extraction of factors for each component*—used to determine the factors that represented the variability of water quality in the Orós reservoir, with the least possible loss of information contained in the total data
(iii) *Extraction of the number of components and communality of each variable*—using the criteria proposed by Kaiser (1958) and Norusis (1990), only components with eigenvalues greater than 1 were considered. The commonality expresses the variance for each variable.
(iv) *Factor transformation*—using studies of Hair Júnior et al. (2005), the varimax orthogonal rotation method was used, in which the attributes are given weights close to 1 or 0, and intermediate values that could make the interpretation difficult are eliminated. Statistical analyses were carried out using SPSS version 16.0 as it is capable of carrying out all the analyses relevant to principal component analysis.

The WQI was calculated as the sum of the individual values of each attribute (*q*_i_) weighted by the importance of this attribute in the evaluation of the total variability of the water quality (*w*_i_). This method has already been successfully used by Almeida and Schwarzbold (2003) and Lopes et al. (2008). The general formula used was

1 $$ WQI = \prod {q_{\mathrm{i}}}^{w\mathrm{i}} $$

where

WQI: water quality index (dimensionless)
Π: multiplier
*q*_i_: relative quality of the *i*th attribute
*w*_i_: relative weighting of the *i*th attribute
i: attribute number

For the limits of *q*_i_ (Table 2), we used the recommendations of water quality for human consumption proposed by Armitage et al. (1983), Junqueira and Campos (1998), Mustow (2002), Lamparelli (2004), Brasil (2004, 2005, 2011), WHO (2006), Boyacioglu (2010), Rubio-Arias et al. (2014) and Rekha et al. (2013). The weighting (*w*_i_) for each attribute of water quality used in the WQI was defined by the component that explained the greatest proportion of the total variance.

**Table 2:** Limits of the attributes used in the WQI for calculation of the *q*_i_

Attribute	Intervals of *q*_i_					
	100–90 (excellent)	90–70 (good)	70–50 (fair)	50–25 (bad)	25–0 (very bad)	References
Ca^2+^ (mg L^−1^)	10 < X <75	75 < X <200	200 < X <500	>500	>500	BRASIL (2004), WHO (2006)
Mg^2+^ (mg L^−1^)	10 < X <30	30 < X <150	150 < X <500	>500	>500	BRASIL (2004), WHO (2006)
Cl^−^ (mg L^−1^)	0 < X <250	250 < X <400	400 < X <600	600 < X <1000	>1000	BRASIL (2004, 2005, 2011), WHO (2006)
Col A uH	0 < X <5	5 < X <10	10 < X <15	15 < X ≤20	20 < X ≤200	BRASIL (2004, 2011)
TSS (mg L^−1^)	0 < X <5	5 < X <15	15 < X <25	25 < X <50	50 < X <100	Boyacioglu (2010)
Tur (uT)	0	0 < X <2.5	2.5 < X <5	5 < X <10	10 < X <100	Brasil (2004, 2011), Rubio-Arias et al. (2014), Rekha et al. (2013)
K + (mg L^−1^)	0 < X <50	50 < X <100	100 < X <200	>200	>200	WHO (2006)
Na^+^ (mg L^−1^)	0 < X <50	50 < X <100	100 < X <200	200 < X <400	400 < X <600	BRASIL (2004,2011), WHO (2006)
SO_4_ (mg L^−1^)	0 < X <100	100 < X <200	200 < X <250	250 < X <1000	>1000	BRASIL (2004,2011), WHO (2006)
ricF (ec) BMWP	≥ 86	64 < X ≤85	37 < X ≤63	17 < X ≤36	≤16	Armitage et al.(1983), Junqueira and Campos (1998), Mustow (2002)
ricF (ps) BMWP	≥ 86	64 < X ≤85	37 < X ≤63	17 < X ≤36	≤16	Armitage et al.(1983), Junqueira and Campos (1998), Mustow (2002)
Total phosphorous (mg L^−1^)	<0.008	0.008 < X <0.019	0.019 < X <0.052	0.052 < X <0.120	0.120 < X <1.2	Lamparelli (2004), Boyacioglu (2010)

*Ca* ^+*2*^ *ion* calcium, *Mg* ^+*2*^ *ion* magnesium, *Cl* ^−^ *ion* chloride, *Col A* apparent Colour, *TSS* total suspended solids, *Tur* turbidity, *K* ^+^ potassium, *Na* ^+*1*^ sodium, *SO*_*4*_ sulphate, *ricF* (*ec*) richness of macroinvertebrate families associated with the roots of the macrophyte *Eichhornia crassipes*, *ricF* (*ps*) richness of macroinvertebrate families associated with the roots of the macrophyte *Eichhornia crassipes*

Definition of the weightings (*w*_i_) assigned to each attribute of water quality used in the WQI was established from the results of the principal component analysis. In this procedure, the eigenvalues of the components, and how much of each attribute was explained by its respective components taken from the PCA, were used. The equation used to calculate *w*_i_ was

2 $$ {w}_{\mathrm{i}}=\frac{\left({F}_1\cdot P{1}_{\mathrm{i}}\right)+\left({F}_2\cdot P{2}_{\mathrm{i}}\right)}{\left({F}_1\cdot {\displaystyle \sum_{j=1}^nP{1}_{\mathrm{j}}}\right)+\left({F}_2\cdot {\displaystyle \sum_{j=1}^nP2\mathrm{j}}\right)} $$

where

*w*_i_: weight assigned to the *i*th variable making up the WQI
*F*_1_ and *F*_2_: eigenvalue of the main components
*P*_i_: explicability of the *i*th variable by principle component
*P*j: explicability of the *j*th variable by principle components
i and j: indices from 0 to 6 for the attributes
*n*: number of variables involved in the PCA

The calculated WQI can have a value between 0 and 100. We divided the values into five classes (Table 3).

**Table 3:** Ranges of water quality for the WQI

Value of WQI	Water quality	Restrictions on use for human consumption^a^
90–100	Excellent	With disinfection
70–90	Good	Simplified treatment
50–70	Fair	Conventional treatment
25–50	Bad	Advanced treatment
0–25	Very Bad	Unsuitable

Comitesinos (1990) and modified by Almeida and Schwarzbold (2003)

*WQI* water quality index

^a^Based on Brasil (2005)

The Student’s *t* test was applied at a significance level of 5 % to compare the means (1) of the WQIs between collection points and (2) for the wet and dry periods.

## Results and discussion

After the factor analysis/principal component analysis (FA/PCA), we were able to bring down the number of attributes from 33 to 12 (Table 4). A test for model adequacy (KMO) resulted in a value of 0.61. The five components explained 29.60, 21.42, 14.20, 11.70 and 8.38 % of the total variance in the data, respectively, with five dimensions explaining 85.32 % of the variance.

**Table 4:** Factor loading matrix of water quality attributes for the Orós reservoir, CE

	Attribute	Component				
		C1	C2	C3	C4	C5
01	Ca^+2^ (mg L^−1^)	0.930	−0.094	0.133	−0.059	0.061
02	Mg^+2^ (mg L^−1^)	0.912	−0.041	0.082	0.044	−0.096
03	Cl^−^ (mg L^−1^)	0.843	0.021	0.253	−0.079	−0.091
04	Apparent colour (uH)	−0.071	0.971	−0.033	0.026	0.074
06	Turbidity (uT)	0.035	0.939	0.131	0.050	0.032
05	Total suspended solids (mg L^−1^)	−0.006	0.902	−0.180	0.096	0.050
07	K^+^ (mg L^−1^)	0.371	−0.054	0.881	−0.062	0.050
08	Na^+^ (mg L^−1^)	0.388	−0.039	0.883	−0.073	0.145
09	SO_4_^−2^ (mg L^−1^)	−0.337	0.007	0.674	0.190	−0.476
10	Richness of the macroinvertebrate family associated with the macrophyte *Eichhornia crassipes*	0.006	−0.046	−0.129	0.837	0.074
11	Richness of the macroinvertebrate family associated with the macrophyte *Pistia stratiotes*	−0.069	0.100	0.104	0.748	−0.173
12	Total phosphorus	−0.148	0.079	0.061	−0.060	0.946
	Eigenvalue	3.55	2.57	1.70	1.40	1.00
	Variance (%)	29.60	21.42	14.20	11.70	8.38
	Accumulated variance (%)	29.60	51.03	65.23	76.94	85.32
	KMO	0.61				

The reduction in attributes with little loss in the explicability of the variance is interesting because it means that the number of laboratory tests can be reduced, thereby saving time and resources (Zeng and Rasmussen 2010); this is especially useful in countries where financial resources are limited (Debels et al. 2012).

Table 4 shows that the principal components C1 and C3 explain 29.6 and 14.20 % of the total variability of the data, respectively, and are associated with the chemical attributes represented by calcium (Ca^+2^, 0.930), magnesium (Mg^+2^, 0.912), chloride (Cl^−^, 0.843), potassium (K^+^, 0.881), sodium (Na^+^, 0.883) and sulphate (S0_4_^−2^, 0.674). These results create two components related to the soluble salts in the water (C1 and C3) that can be explained by the presence of crystalline and carbonate rocks of the Orós group that are found in the Upper Jaguaribe watershed.

These salts may be related to the weathering process, as verified by Meireles et al. (2007) and Andrade et al. (2007), and to domestic sewage inputs (Pal et al. 2013). Evaporation also contributes negatively to water quality in reservoirs and is aggravated by the lack of water renewal in the dry season (Palácio et al. 2011).

Similarly, Deepak and Singh (2013), in studies conducted in Dhar, India, claim that the high levels of salts in water bodies are related to inputs of domestic and industrial effluents. Kumar and James (2013) also noted that increases in these elements are due mainly to industrial and agricultural activities and to the lack of basic sanitation.

The second component, C2, explains 21.42 % of the variability in the data and is associated with the physical attributes of apparent colour (0.971), total suspended solids (0.902) and turbidity (0.939), which basically reflect surface runoff. These results suggest that the reservoir has been receiving a large input of suspended matter from soil erosion and degradation of the riparian vegetation, residues of agricultural fertilisers, and excessive loads of domestic sewage and solids disposed of improperly on the reservoir shores (Lopes et al. 2014).

Batista et al. (2014) carried out an evaluation of the trophic state of the Orós reservoir and concluded that sediments transported by surface runoff played a key role in reducing water transparency and that there was seasonal variation in the trophic status of the reservoir waters. Trophic levels are higher in the dry season because of the lower volume of stored water.

These findings are consistent with those from other studies carried out by Lopes et al. (2014) and Santos et al. (2014) in the Orós reservoir, in which they point to weathering, surface runoff and human activity as being responsible for most deterioration in water quality. These authors suggest that intervention is necessary in order to reduce inputs of waste, which would then improve the trophic state of the water in the reservoir.

Table 4 also shows that component C4 explains 11.70 % of the total variability in the data. C4 is related to the biological attributes, as represented by the richness of families associated with the aquatic macrophytes *E. crassipes* (0.837) and *P. stratiotes* (0.748) (Table 5).

**Table 5:** Macroinvertebrate family richness and scores assigned to each family

Level of macroinvertebrates			Abundance		Score^a^
Class	Order	Family	*Pistia stratiotes*	*Eichhornia crassipes*	
Malacostraca	Decapoda	Atydae	198	71	8
Insecta	Odonata	Libellulidae	31	17	5
		Perilestidae	93	44	5
	Coleoptera	Dyticidae	19	45	5
		Hydrophilidae	97	85	4
		Elmidae	19	53	4
	Diptera	Chironomidae	6	22	2
Gastropoda	Mesogastropoda	Ampularidae	1	36	3
	Neotaenioglossa	Thiaridae	823	1550	3
	Basommatophora	Planorbidae	207	256	3
Abundance			2179	1494	

^a^BMWP and adaptations

Three classes and eight orders were found (Decapoda, Araneae, Odonata, Coleoptera, Diptera, Mesogastropoda, Neotaenioglossa and Basommatophora) distributed over ten families (Atyidae, Libellulidae, Perilestidae, Dyticidae, Hydrophilidae, Elmidae, Chironomidae, Ampularidae, Thiaridae and Planorbidae). All of the families were associated with the two species of aquatic macrophytes. The presence of these families is clear evidence of environments with poor water quality (Wyżga et al. 2013).

Results from the biological component showed that resistant families were present at all of the sampling points. Results were highest at point P2, showing that the environmental conditions at this site are unfavourable to families that are sensitive to environmental changes. These results are consistent with those of Ngodhe et al. (2014) and Ogrena and Huckins (2014), who also used macroinvertebrate biological metrics as indicators of water quality. Ogrena and Huckins (2014), in their studies of the Manistee watershed, found that bioindicators responded accurately to the quality of the local water.

The low diversity and families of macroinvertebrates, and in particular the Thiaridae family, associated with the two species of macrophyte are indicators of poor water quality.

Integrating biological attributes of macroinvertebrates with physical and chemical attributes made it possible to distinguish the degree of water quality deterioration at the different sampling points of the Orós reservoir; it also indicated the main agents responsible for the loss of environmental quality. Results confirm that the macroinvertebrate community is sensitive to changes in the aquatic environment.

Similar results were obtained by Mustow (2002) in biomonitoring studies carried out in Thai rivers. The author defends the inclusion of macroinvertebrate metrics, stating that they provide a rapid means for assessing water quality, with the added benefit of significantly lower costs.

Piedras et al. (2006) reported that the deterioration of water quality resulted in low macroinvertebrate diversity and that it also prevented the development of certain macroinvertebrate groups. Melo and Hepp (2008) also considered that biological metrics of richness, abundance and uniformity were capable of providing relevant information about the conservation of water bodies and that impacted environments tend to have limited biological diversity, with only a few dominating species.

Component C5 explains 8.38 % of the total variance and is related to a single nutrient, total phosphorous. C5 has a weighting of 0.946 (Table 4) and is an indicator of phosphorus enrichment. Phosphorus-rich waters favour the development of algae and the consequent eutrophication of the aquatic environment. Rabee et al. (2011), in studies conducted on the River Tigris in Iraq, found that phosphorus was the main factor responsible for eutrophication of the river and that it contributed to the excessive proliferation of microalgae in water bodies. Guedes et al. (2012) also observed drinking water quality deterioration due to phosphorus in the River Pomba in Minas Gerais, as did Silva (2013) in his study of phytoplankton communities in the Orós reservoir.

Other factors, such as fish farming and evaporation, may be related to increases in nutrients and consequent decreases in water quality. Mallasen et al. (2012), in their study of water quality in the Orós reservoir, found that phosphorus enrichment was due to an increase in fish rearing using net cages, because the feed for the fish is very rich in phosphorus. We can therefore assume that the water quality of the Orós reservoir is strongly influenced by the nature of the rocks and soil types in the region, and by anthropogenic activities that promote nutrient inputs to water bodies (livestock, fish farming and the erosion of agricultural areas).

The largest weightings for the WQI were recorded by attributes related to water hardness (Table 6). The higher factor weighting values indicate the most significant attributes for each factor. Ca^+2^, Mg^+2^ and Cl^−^ had the highest weightings (>0.10); the index displays high sensitivity to variability of these ions. In contrast, the biological attributes related to the richness of macroinvertebrate families and to total phosphorus had lower weightings, indicating little sensitivity to variations in these attributes.

**Table 6:** Weightings (*w*_i_) for the respective attributes of the WQI

Attribute	Weighting
Calcium (Ca^+2^)	0.113
Magnesium (Mg^+2^)	0.106
Chloride (Cl^−^)	0.105
Apparent colour	0.093
Total suspended solids	0.090
Turbidity	0.089
Potassium (K^+^)	0.084
Sodium (Na^+^)	0.083
Sulphate (SO_4_^−2^)	0.082
Richness of the family associated with *Eichhornia crassipes*	0.055
Richness of the family associated with *Pistia stratiotes*	0.054
Total Phosphorus	0.046
Total	1.000

Similarly, Andrade et al. (2005) found that weightings were highest for attributes related to the water salt concentration (Na^+^, sodium absorption ratio (SAR) and EC), and lower weightings were related to the presence of organic compounds, pH and NO_3_^−^. In their study of water quality in the River Odzi, Jonnalagadda and Mhere (2001) found that weightings were highest for pH and BOD.

### Values of WQI for surface water in the Orós reservoir

Mean values of the attributes chosen to represent the water quality of the reservoir are presented in Table 7. It is noteworthy that the mean values for Ca^+2^, Mg^+2^, Cl^−^, total suspended solids, K^+^, Na^+^ and SO_4_^−2^ are within the acceptable limits for human consumption. Colour, turbidity, total phosphorus and macroinvertebrate family richness associated with the two aquatic macrophytes were considered unfit for human consumption, based on the limits suggested by Brazilian and international legislation (Table 2).

**Table 7:**
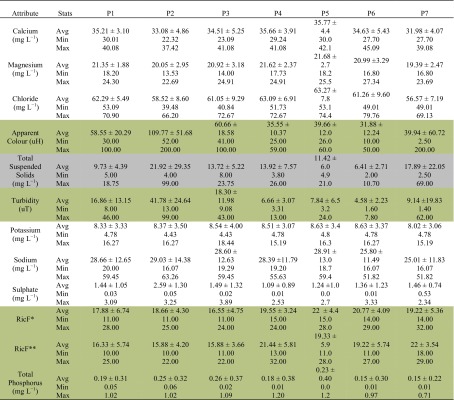
Statistics of water quality attributes of the Orós reservoir

White background: excellent quality; light grey: good quality; light brown: very bad

RicF* richness of the macroinvertebrate family associated with the aquatic macrophyte *Eichhornia crassipes*, RicF** richness of the macroinvertebrate family associated with the aquatic macrophyte *Pistia stratiotes*

Table 8 shows the values of *q*_i_ calculated from the results of the attribute analyses. Table 9 shows that values of the WQI at the sampling points are similar, indicating that there was little spatial or temporal variability in the water quality of the Orós reservoir (ts = 0.001, *p* > 0.05). Furthermore, there were no significant differences between the dry or rainy seasons (ts = 0.001, *p* > 0.05).

**Table 8:** Water quality index values for surface waters of the Orós reservoir

Collection	P1	P2	P3	P4	P5	P6	P7	Avg.	C.V.
August 2009	61.49	59.08	57.71	59.98	61.09	65.55	62.22	61.02	4.12
October 2009	56.70	54.18	55.43	62.62	65.85	67.38	67.95	61.44	9.63
December 2009	55.68	41.21	52.94	63.31	65.40	64.06	65.57	58.31	15.49
February 2010	57.48	43.89	58.98	58.85	57.11	64.69	51.6	56.08	11.78
April 2010	56.85	52.67	55.99	67.02	65.58	63.68	68.09	61.41	9.98
June 2010	47.03	50.15	48.56	41.24	44.97	53.68	60.85	49.50	12.84
September 2010	52.64	55.00	51.84	64.64	60.69	65.80	79.94	61.51	16.03
January 2011	47.15	51.00	48.89	61.09	52.46	69.12	72.73	57.49	17.80
March 2011	58.92	42.29	55.88	62.10	61.48	65.33	62.96	58.42	13.22
Average	54.88	49.94	54.02	60.09	59.40	64.37	65.76		
C.V.	9.13	12.40	6.85	12.44	11.70	6.75	12.08		

*C.V.* Coefficient of Variation

**Table 9:** Comparison of average WQI values for the rainy and dry seasons for the Orós reservoir, CE

Sampling point	Statistic	Season	
		Dry	Rainy
P1	Average	54.70 ± 5.34	55.09 ± 5.37
	C.V.	9.76	9.74
P2	Average	51.92 ± 6.77	47.46 ± 10.82
	C.V.	13.05	25.54
P3	Average	53.29 ± 3.49	54.93 ± 7.78
	C.V.	6.55	7.78
P4	Average	58.35 ± 9.71	62.26 ± 5.53
	C.V.	16.65	5.53
P5	Average	59.60 ± 8.51	59.15 ± 9.54
	C.V.	14.29	9.54
P6	Average	63.29 ± 5.50	65.70 ± 3.61
	C.V.	8.69	3.61
P7	Average	67.30 ± 7.59	63.84 ± 14.23
	C.V.	11.28	26.93
	Average	58.35 ± 5.57	58.35 ± 3.63
	C.V.	9.54	8.66

*C.V.* Coefficient of Variation

The mean WQI values at the sampling points ranged from 49 to 65 (rated as normal), indicating that water is suitable for human consumption as long as the water is treated. Water treatments proposed for the waters of the Orós reservoir follow the recommendations of Brasil (2005) and involve removal and/or inactivation of refractory constituents that influence the colour, odour, taste, toxicity or pathogenic activity by conventional treatment processes.

Almeida and Schwarzbold (2003) applied the NSF WQI to the Cria Montenegro Stream (RS) and found that water quality was low. Andrade et al. (2005) obtained WQI values of between 72 and 89 for the Trussu River Valley, CE, which indicate that the water may be used for human consumption. Lopes et al. (2008) also applied a WQI to the River Acaraú and obtained values ranging from 60 to 80. Franco and Hernandez (2012), in their study of water quality in the Coqueiro catchment in São Paulo, obtained values ranging from 38 (acceptable) to 92 (excellent). Melo Junior et al. (2003), found values ranging from 59 to 85 (fair to good) for a stretch of the River Açu, in Rio Grande do Norte.

WQI values were highest at sampling points P7 (65.76) and P6 (64.37), located near the spillway of the reservoir. These high values highlight the purification capacity of the reservoir (Ostroumov 2005; Wei et al. 2009), as these points are furthest from the sediment and nutrient inputs from runoff.

Water quality was worst at P2 (49.94), P3 (54.02), P1 (54.88), P5 (59.40) and P4 (60.09); these sampling points are located at the upper end of the reservoir. The low results are mainly due to the very high values for apparent colour, turbidity and total phosphorus, and to the low levels of macroinvertebrate family richness. Sampling point P2 is located close to the inflow of the River Jaguaribe, the largest tributary with the largest nutrient inputs. The apparent colour, turbidity, macroinvertebrate richness and total phosphorus contribute the most to water quality deterioration.

Inputs of sewage and household waste from the town of Iguatu, approximately 20 km from the reservoir, are thought to make a significant contribution to the contamination of the Orós reservoir, and, in particular, upstream of sampling point P2. Iguatu has a population of 96,495, and data from IPECE (2014) indicate that only 11.25 % of the population is connected to an urban sewage system. Batista et al. (2014) agreed and classified P2 as hypereutrophic, and P1 and P3 as supereutrophic, due to the low values for transparency, and high total phosphorus levels. Overall, the reservoir was classified as hypereutrophic because of sediments carried by surface runoff.

Using the WQI, water quality is classified as fair, meaning that it can be used for human consumption. However, it should be noted that not all the attributes included in Table 7 are within acceptable limits. This suggests that the WQI should be adapted in order to detect the sensitivity of attributes such as total phosphorus and macroinvertebrate communities.

## Conclusions

1. Using factor analysis/principal component analysis, we reduced the number of water quality attributes from 33 to 12; these 12 attributes explained 85.32 % of the total variance.
2. The five components from factor analysis/principal component analysis highlighted that weathering runoff and nutrient inputs, resulting from human activities such as agriculture, livestock, sewage discharge and household waste, were the main factors responsible for water quality deterioration in the Orós reservoir.
3. The proposed WQI, based on physical and chemical attributes and a macroinvertebrate metric, showed that water quality in the Orós reservoir is classified as ‘fair’, meaning that it is suitable for human consumption, as long as advanced treatment is carried out.
4. WQI values were highest at points P7 and P6, located near the spillway of the reservoir, reflecting the purification capacity of the reservoir.
5. The worst water quality was recorded at points P2, P3, P1, P4 and P5; these sampling points are located at the upper end of the reservoir, and the poor water quality reflects nutrient inputs, especially total phosphorus.
6. This study presents a more holistic view of the water quality of the Orós reservoir due to the inclusion of macroinvertebrate metrics in the physical and chemical attributes.
7. The low diversity of macroinvertebrates associated with the two species of macrophytes, and the dominance of pollution-resistant families indicate degradation of the water quality of the Orós reservoir.
